# Supplementary material for: Thermoelectric Properties of N-Type Poly (Ether Ether Ketone)/Carbon Nanofiber Melt-Processed Composites
Source: Polymers (Basel). 2022 Nov 8;14(22):4803. doi: 10.3390/polym14224803 (PMC9699565; doi:10.3390/polym14224803)
Supplement: Supplementary file 1 [file polymers-14-04803-s001.zip › polymers-1984596-supplementary.pdf]

polymers

Supporting Information

Thermoelectric Properties of N-Type Poly (Ether Ether Ketone)/Carbon Nanofiber Melt-Processed Composites

Antonio Jose Paleo [1,*], Beate Krause [2], Delfim Soares [3], Manuel Melle-Franco [4], Enrique Muñoz [5], Petra Pötschke [2] and Ana Maria Rocha [1]

1 2C2T-Centre for Textile Science and Technology, Campus Azurem, University of Minho, 4800-058 Guimarães, Portugal
2 Leibniz-Institut für Polymerforschung Dresden e.V. (IPF), Hohe Str. 6, 01069 Dresden, Germany
3 CMEMS Research Center, Campus Azurem, University of Minho, 4800-058 Guimarães, Portugal
4 CICECO—Aveiro Institute of Materials, Department of Chemistry, University of Aveiro, 3810-193 Aveiro, Portugal
5 Facultad de Física, Pontificia Universidad Católica de Chile, Santiago 7820436, Chile
* Correspondence: ajpaleovieito@2c2t.uminho.pt

Table S1. Thermoelectric properties of carbon nanotube and carbon nanofiber powders and their melt-processed PEEK composites.

Sample	Filler content	Conductivity	Seebeck Coefficient	Power Factor	Reference
	wt. %	S m^{-1}	μV K^{-1}	μW·m^{-1}·K^{-2}	
SWCNT Tuball™	100 (powder)	1790	39.6	2.8	1
MWCNT Nanocyl™ NC7000	100 (powder)	417	6.3	1.6×10^{-2}	1
MWCNT CNS-PEG	100 (powder)	933	10.1	9.5×10^{-2}	1
CNF Pyrograf® III PR 24 LHT XT	100 (powder)	133.5	-5.3	3.7×10^{-3}	This work
CNF Pyrograf® III PR 19 LHT XT	100 (powder)	136.4	-5.1	3.5×10^{-3}	3
PEEK-Tuball	0.5 -1.25	1.8-7.2	48.0 -61.3	6.6×10^{-3}-2.0 $\times 10^{-2}$	2
PEEK-Nanocyl® NC7000	3; 5	5.2; 51.7	7.3; 6.8	2.7×10^{-4} 2.3 $\times 10^{-3}$	2
PEEK- Nanocyl™ NC 7000	3; 4	1.1; 12.4	9.7; 9.2	1.0×10^{-4} 1.0×10^{-3}	4
PEEK-CNS-PEG	0.5 – 3	8.8-97.1	13.4-16.1	1.8×10^{-3} 2.1 $\times 10^{-2}$	2
PEEK- CNF Pyrograf® III PR 24 LHT XT	10	27.5	-3.4	3.1×10^{-4}	This work

References:

1. Beate Krause, Carine Barbier, Juhasz Levente, Maxim Klaus, Petra Pötschke, Screening of different carbon nanotubes in melt-mixed polymer composites with different polymer matrices for their thermoelectrical properties, Journal of Composites Science 2019, 3(4), 106; doi:10.3390/jcs3040106
2. Ioannis Konidakis, Beate Krause, Gyu-Hyeon Park, Nithin Pulumati, Heiko Reith, Petra Pötschke, Emmanuel Stratakis, Probing the carrier dynamics of polymer composites with single and hybrid carbon nanotube fillers for improved thermoelectric performance, ACS Applied Energy Materials 2022, 5, 9770–9781, doi:10.1021/acsaem.2c01449.

3. A.J. Paleo, B. Krause, M.F. Cerqueira, E. Muñoz, P. Pötschke, A.M. Rocha, Nonlinear Thermopower Behaviour of N-Type Carbon Nanofibres and Their Melt Mixed Polypropylene Composites, Polymers, 14 (2022). Doi:10.3390/polym14020269.
4. Gonçalves, Jordana; Lima, Patrícia; Krause, Beate; Pötschke, Petra; Lafont, Ugo; Gomes, José ; Abreu, Cristiano ; Paiva, Maria ; Covas, José, Electrically Conductive Polyetheretherketone Nanocomposite Filaments: From Production to Fused Deposition Modeling, Polymers 2018, 10 (8), 925, https://doi.org/10.3390/polym10080925
